# A low-redox-potential phenazine-based negolyte with high stability for aqueous organic flow batteries

**DOI:** 10.1039/d5sc07193c

**Published:** 2025-10-23

**Authors:** Xuanyu Xie, Taoyi Kong, Jiaming Gao, Ruiyang Li, Yonggang Wang

**Affiliations:** a Department of Chemistry, Shanghai Key Laboratory of Molecular Catalysis and Innovative Materials, Institute of New Energy, iChEM (Collaborative Innovation Center of Chemistry for Energy Materials), Fudan University Shanghai 200433 China ygwang@fudan.edu.cn

## Abstract

Phenazine-based negolytes for aqueous organic redox flow batteries (AOFBs) have attracted significant attention due to their structural tunability and highly reversible redox behaviour. However, phenazine derivatives often suffer from adverse tautomerization and side-chain cleavage and their stability would become inferior as redox potential decreases. Consequently, developing stable phenazine derivatives with low redox potential (especially ≤−0.8 V *vs.* SHE) remains a major challenge, yet it is essential for achieving high-voltage AORFBs. Herein, we report a new phenazine derivative, 4,4′-((7,8-dimethoxyphenazine-2,3-diyl)bis(oxy))dibutyric acid (dMeODBAP), which exhibits a favourable redox potential of −0.84 V (*vs.* SHE) along with exceptional stability, as validated by both spectroscopic analyses and theoretical simulations. Besides, a microporous blend membrane is fabricated, which effectively suppresses the crossover issue and exhibits a higher potassium ion conductivity than the commercial Nafion 212 membrane. Hence, the AOFB based on dMeODBAP and the blend membrane achieves a high voltage of 1.34 V, an impressive peak power density of 194 mW cm^−2^ and a remarkable capacity retention of 99.95% over 1000 cycles at 100 mA cm^−2^, corresponding to an ultra-low capacity fade rate of 0.007% per day.

## Abstract 2

A stable phenazine-based negolyte (dMeODBAP) with a redox potential as low as −0.84 V (*vs.* SHE) is developed. Hence, the dMeODBAP-based AOFB shows a high voltage of 1.34 V and good stability corresponding to a low fade rate of 0.007% per day.

## Graphical abstract

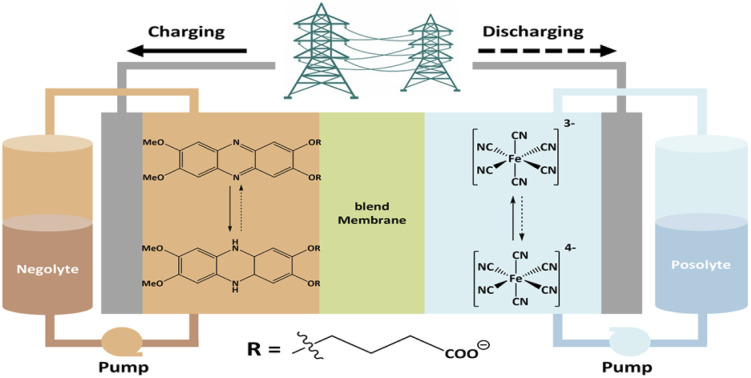

## Introduction

In recent years, the environmental problems caused by the excessive use of fossil energy have become increasingly serious.^1^ In order to achieve sustainable development, the rational utilization of renewable energy sources has aroused a lot of attention. However, the intermittent and volatile nature of these sources impedes their applications in large-scale grids. To address this issue, high-efficiency energy storage devices are required to achieve reliable renewable integration and balance the mismatch between supply and demand.^2^ Aqueous organic flow batteries (AOFBs) utilizing electroactive organic molecules as active species have emerged as promising candidates due to their intrinsic safety, high scalability and independent control over energy and power.^3–7^ So far, AOFBs utilizing anthraquinones^8–23^ or phenazines^24–33^ as active materials in negolytes have been extensively studied because of their desirable properties, including highly reversible redox behaviour and environmental sustainability.^34,35^

Phenazine-based negolytes reported previously often suffer from adverse side reactions such as tautomerization and side chain cleavage. And their stability would become inferior as redox potential decreases.^25^ To date, lots of efforts have been made to raise the stability of phenazine-based negolytes with moderate potentials above −0.8 V (*vs.* SHE). For example, research by Liu *et al.* has demonstrated that introducing long-chain substituents into the phenazine core is an effective method to mitigate the undesired side chain cleavage.^30^ Xu *et al.* confirm the superior stability of phenazine compounds modified with carbon-linked functional groups even at elevated temperature.^28^ In addition, phenazine compounds substituted at the 2,3-positions would not undergo tautomerization thermodynamically and therefore exhibit exceptional stability according to the study by our group.^29^ However, stable phenazine-based negolytes with potentials lower than −0.8 V (*vs.* SHE) are highly desired to enable long-life and high-energy-density AOFBs. Unfortunately, it still remains a considerable challenge to endow low-redox-potential phenazine derivatives with high stability.^25^

In this work, we report a new phenazine derivative, 4,4′-((7,8-dimethoxyphenazine-2,3-diyl)bis(oxy))dibutyric acid (dMeODBAP). The accumulated electron-donating effect of substituents reduces the redox potential of dMeODBAP to as low as −0.84 V (*vs.* SHE). This value is highly competitive among analogous molecules and is beneficial for enhancing the open-circuit voltage of the full battery. Additionally, as many as four substituents are introduced into the phenazine core, which endows dMeODBAP with a relatively large molecular size and effectively suppresses the capacity fade caused by the crossover issue. Furthermore, the exceptional chemical stability and electrochemical reversibility of dMeODBAP is confirmed by both experimental characterizations and theoretical calculations. Besides, a microporous blend membrane is fabricated, which effectively prevents the dMeODBAP from crossing the membrane and also exhibits a higher potassium ion conductivity than the commercial Nafion 212 membrane. Hence, the AOFB based on dMeODBAP and the blend membrane delivers a high voltage of 1.34 V and an output power density of 194 mW cm^−2^, and exhibits a remarkable capacity retention of 99.95% for 1000 cycles at 100 mA cm^−2^, corresponding to an ultra-low daily capacity fade rate of 0.007%. A cell is also fabricated with the Nafion 212 membrane for comparison, which shows a lower capacity retention (94% for 1000 cycles at 100 mA cm^−2^). The capacity decay of the AOFB based on Nafion 212 is attributed to the adsorption of dMeODBAP on the membrane, which increases the resistance and degrades capacity utilization.

## Results and discussion

**Fig. 1:**
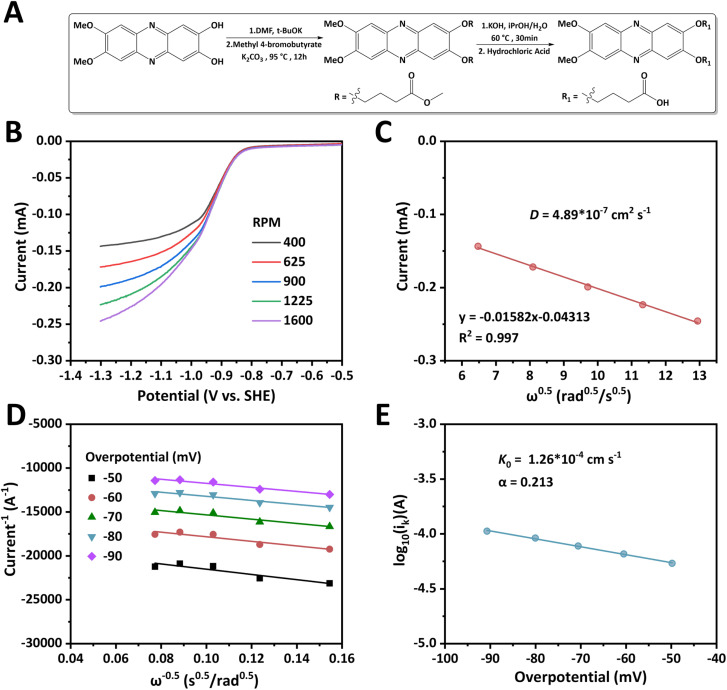
Synthesis and redox kinetics study of dMeODBAP. (A) A scheme showing the synthesis route of dMeODBAP. (B) LSV curves at a scan rate of 10 mV s^−1^ at different rotation rates. (C) Levich plot of the limiting currents *vs.* the square roots of rotation rates. (D) Koutecký–Levich plots at different overpotentials, respectively. (E) Fitting plot of the Butler–Volmer equation at different overpotentials.

Fig. 1A showcases part of the synthetic route of dMeODBAP and the details are provided in the SI. First, 1,2-dimethoxybenzene is nitrated by nitric acid and the obtained 1,2-dimethoxy-4,5-dinitrobenzene is reduced by hydrazine hydrate. The product, 4,5-dimethoxybenzene-1,2-diamine, is then reacted with 2,5-dihydroxy-1,4-benzoquinone to obtain 7,8-dimethoxyphenazine-2,3-diol, which is then mixed with methyl 4-bromobutyrate, anhydrous potassium carbonate and potassium *tert*-butoxide in dimethylformamide and heated at 95 °C for 12 hours. The solution is then poured into ice water and filtered to obtain an ester. The ester is hydrolyzed by potassium hydroxide and adjusted to pH 4 with hydrochloric acid to obtain the final dMeODBAP. ^1^H NMR was performed to verify the molecular structure of the intermediates and the dMeODBAP (Fig. S1–S3). In particular, dMeODBAP exhibits a bulky molecular size due to its multi-substituted structure. If the external contour of dMeODBAP is simplified to a rectangle, the length of its short side would reach 12.7 Å (Fig. S4).

In addition, we fabricated a microporous blend membrane according to a method reported by Song *et al.*^36^ A block copolymer, TTB-PIM-COOH, was firstly prepared and its structure was investigated by studying its ^1^H NMR spectrum (Fig. S5). And then, TTB-PIM-COOH was physically blended with the previously reported AO-PIM-1 to obtain the final blend membrane. The pore size distribution curve showcases a predominance of subnanometer-sized micropores in the blend membrane, especially in the narrow range of 3 Å to 7 Å (Fig. S6). These results indicate that the molecular size of dMeODBAP surpasses the vast majority of the pores' size in the blend membrane. A bulky size is beneficial for mitigating the capacity degradation caused by the crossover of dMeODBAP through the membrane, which is confirmed by the subsequent permeation and long-term cycling tests.

UV-vis spectra were recorded at different pH values to reveal the dissociation behaviour of carboxyl groups in dMeODBAP (Fig. S7). As the pH value decreases, the intensity of the light absorbance peak at 254 nm weakens and its shape becomes broader. The light absorbance at 254 nm *versus* pH plot demonstrates that the p*K*_a_ value of dMeODBAP is around 5.3, which indicates that two carboxyl groups dissociate *via* one step. More importantly, the results also demonstrate that the solubility of dMeODBAP would be higher, enabled by the dissociation of carboxyl groups above pH 5.3, especially under strongly alkaline conditions. Therefore, the chemical, physical and electrochemical properties of dMeODBAP were investigated at pH 14. Hence, a ^1^H NMR test was carried out to determine the solubility of dMeODBAP in 1 M KOH solution and the value was measured to be 0.25 M (Fig. S8). In addition, to investigate the molecule's proton and electron-transfer behaviour during the redox processes, CV measurements at various pH values were carried out (Fig. S9). According to the Pourbaix diagram, the relevant slope for dMeODBAP was calculated to be −57.5 mV pH^−1^ (compared to the theoretical value of −59.2 mV pH^−1^), which is in line with the two-electron/two-proton process.

Linear sweep voltammetry (LSV) was performed on a glassy carbon rotating disk electrode to determine the diffusion coefficient (*D*) of dMeODBAP and its kinetic reaction rate constant (*k*_0_) in 1.0 M KOH. The LSV curves at 10 mV s^−1^ under various rotation rates between 400 and 1600 rpm are presented in Fig. 1B. By linearly fitting the limiting diffusion currents and the square root of the rotation rates according to the Levich equation, the diffusion coefficient was calculated to be 4.89 × 10^−7^ cm^2^ s^−1^ (Fig. 1C). In the overpotential region between 50 and 90 mV, a series of plots are depicted according to the Koutecky–Levich equation and kinetic current (*i*_k_) is obtained (Fig. 1D). The linear fitting relationship between the logarithm of *i*_k_ and the overpotential was in accordance with the Bolter–Volmer equation (Fig. 1E). The standard rate constant (*k*_0_) was calculated to be 1.26 × 10^−4^ cm s^−1^.

**Fig. 2:**
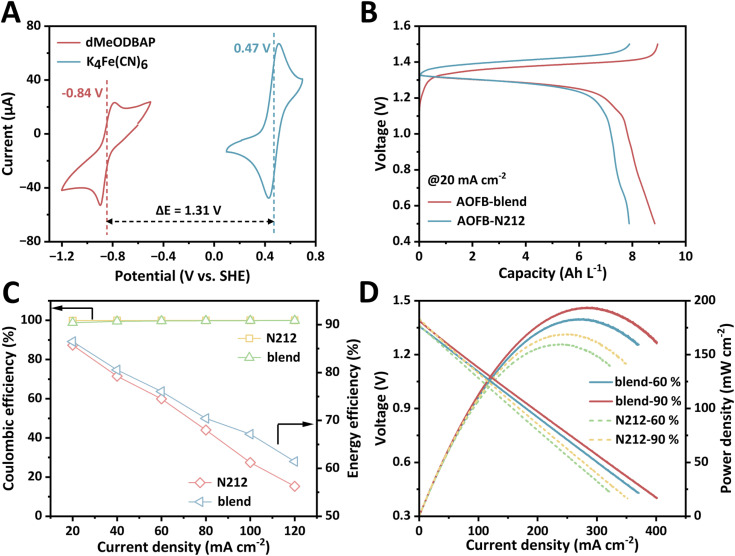
The redox activity of dMeODBAP. (A) CV curves of 5 mM dMeODBAP and 10 mM K_4_Fe(CN)_6_ dissolved in 1 M KOH solutions, respectively. (B) Charge and discharge curves of AOFB-blend and AOFB-N212 under 20 mA cm^−2^. (C) Coulombic efficiency and energy efficiency at different current densities for AOFB-blend and AOFB-N212. (D) Cell voltage and power density *versus* current density at different SOC for AOFB-blend and AOFB-N212.

Cyclic voltammetry (CV) was carried out for the analysis of the electrochemical properties of dMeODBAP in 1.0 M KOH aqueous solution (Fig. 2A and S10). The dMeODBAP molecule exhibits a redox potential as low as −0.84 V (*vs.* SHE), which represents one of the lowest values among negolytes for alkaline AOFBs (Table S1). This impressively low value is attributed to the accumulated electron-donating effect of methoxy groups as well as ether-linked alkyl chains. Apart from the redox potential, its redox reversibility is also illustrated by the anodic/cathodic peaks' separation of 100 mV. Fig. 2A showcases the comparison of the CV curves of dMeODBAP and K_4_Fe(CN)_6_. As the redox potential of dMeODBAP differs by 1.31 V from that of K_4_Fe(CN)_6_ (0.47 V *vs.* SHE), a full cell assembled with dMeODBAP as the negolyte and K_4_Fe(CN)_6_ as the posolyte is anticipated to exhibit an open-circuit voltage exceeding 1.3 V. In order to explore the electrochemical properties of dMeODBAP in detail, we assembled a dMeODBAP‖K_4_Fe(CN)_6_ full cell utilizing the blend membrane (denoted as AOFB-blend) and investigated its working performances under various current densities. Meanwhile, a full cell utilizing Nafion 212 (denoted as AOFB-N212) was assembled and investigated for comparison. As shown in Fig. 2B, both cells deliver high discharge voltages of around 1.3 V, which validates the CV prediction in Fig. 2A.

Interestingly, at the current density of 20 mA cm^−2^, a notable discrepancy was observed in the average charge voltages of the two batteries (1.37 V *vs.* 1.41 V) (Fig. 2B). We suppose that this discrepancy in charge voltages is mainly attributed to the different resistance of AOFB-blend and AOFB-N212 during charging. A possible mechanism is that during charging the negatively charged dMeODBAP migrates towards the posolyte forced by the electric field and finally comes into contact with the membrane. Fortunately, the size of dMeODBAP is considerably bulky, whose short side has a length of 12.7 Å, making it hardly enter the subnanometer pores (3 Å to 7 Å mostly) of the blend membrane and avoiding an increase in resistance. In contrast, dMeODBAP could enter the flexible ion transport regions in Nafion 212 more easily and therefore increase the resistance. This assumption will be further confirmed by the subsequent permeation and long-cycling test.

Electrochemical impedance spectroscopy (EIS) measured under open-circuit voltage (OCV) conditions showcases that the high-frequency area-specific resistance (ASR) of AOFB-blend is lower than that of AOFB-N212 (1.77 Ω cm^2^*vs.* 2.19 Ω cm^2^) (Fig. S11). Furthermore, as the current density increases, AOFB-blend exhibits higher energy efficiency than AOFB-N212, while its coulombic efficiency remains nearly 100% (Fig. 2C). The charge–discharge curves under varying current densities from 20 to 120 mA cm^−2^ are presented in Fig. S12. In addition, the polarization curves of the two AOFBs were collected using the LSV technique at state-of-charge (SOC) levels of 60% and 90% (Fig. 2D). Due to the low ASR, AOFB-blend displayed a peak power density of 194 mW cm^−2^ at 90% SOC, which is superior to that of AOFB-N212 (168 mW cm^−2^).

**Fig. 3:**
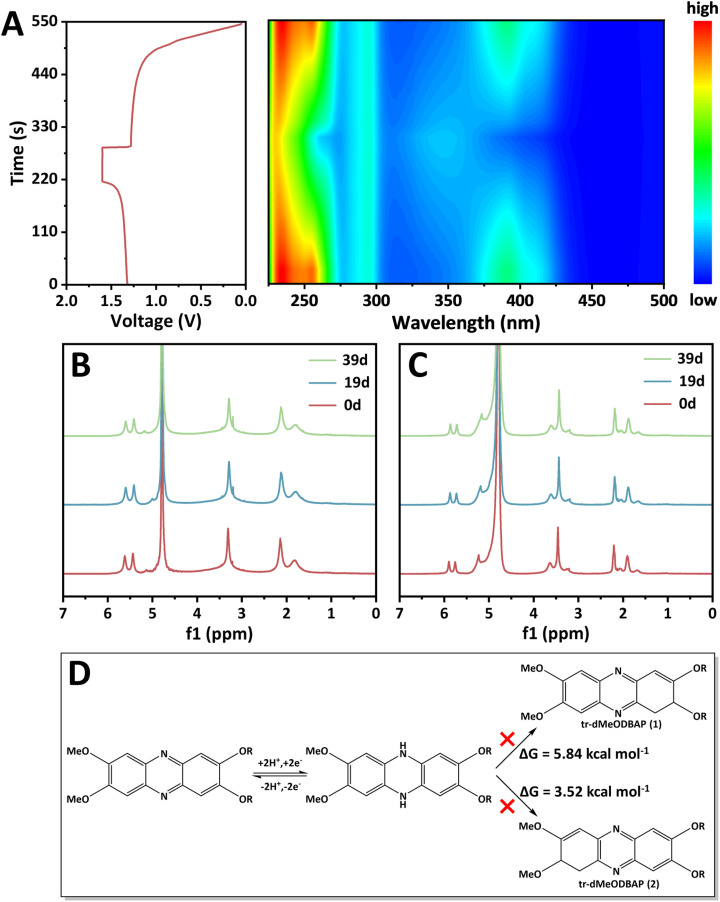
Redox reversibility and chemical stability of dMeODBAP. (A) Voltage–time curve and corresponding 2D color filled contour plot of the *in situ* UV-vis test. (B) Time-dependent ^1^H NMR spectroscopy data for 0.2 M dMeODBAP in 1 M KOH solution. (C) Time-dependent ^1^H NMR spectroscopy data for 0.2 M re-dMeODBAP in 1 M KOH solution. (D) The investigation of potential tautomerization pathways of dMeODBAP by DFT calculations.

Redox reversibility and chemical stability are also critical indicators for the evaluation of electroactive molecules. The redox behaviour of dMeODBAP during the cycling process was investigated by *in situ* UV-vis absorption (Fig. 3A). The original colour of the negolyte was dark brown and transformed into blue upon charging to its reduced form. As the charging process continued, the absorption peak at λ_254nm_ became broader and its peak intensity declined. At around 100% SOC, it shifted from 254 to 247 nm and this blue shift phenomenon indicated a larger HOMO–LUMO energy gap of reduced dMeODBAP. The changes in absorption peaks at various SOC are shown in Fig. S13, demonstrating the good redox reversibility of dMeODBAP. Also, to the best of our knowledge, the tautomerization of phenazine derivatives' reduced state from electroactive to electroinactive species is the main cause of capacity fade.^27^ Given this, time-dependent ^1^H NMR spectroscopy was performed to investigate the chemical stability of dMeODBAP and its reduced state (Fig. 3B and C, respectively). 0.2 M dMeODBAP was dissolved in D_2_O to prepare a negolyte and charged in a galvanostatic–potentiostatic pattern to obtain a solution of re-dMeODBAP. Samples taken before and after charging were stored in an Ar-filled glovebox and ^1^H NMR measurements were carried out for various time intervals. After a 39-day period, the NMR spectra of dMeODBAP and re-dMeODBAP remained unchanged, demonstrating almost no chemical deposition. Furthermore, DFT calculations were performed to evaluate the chemical stability of the reduced state of dMeODBAP (re-dMeODBAP) by comparing the energies of re-dMeODBAP and its potential tautomers (tr-dMeODBAP (1) and tr-dMeODBAP (2)). The tautomerization energies (Δ*G*) for the conversion from re-dMeODBAP to the two tr-dMeODBAP isomers are calculated to be 5.84 and 3.52 kcal mol^−1^, respectively (Fig. 3D and Table S2). These results indicate that the conversion is not a thermodynamically spontaneous process, confirming the conclusion of time-dependent ^1^H NMR tests.

Besides, a low permeability of electroactive molecules is critical for mitigating the capacity fade caused by the crossover issue. An H-cell was used to measure the permeability of dMeODBAP across two types of membranes used in this work. As shown in Fig. S14, the permeability of dMeODBAP across Nafion 212 and the blend membrane is measured to be 5.03 × 10^−14^ and 8.27 × 10^−14^ cm^2^ s^−1^, respectively. These low values indicate that dMeODBAP in the negolyte side can hardly pass through the pores of the blend membrane into the posolyte side. Although the permeability of dMeODBAP across Nafion 212 is also very low, the advantage of the blend membrane over Nafion 212 lies in its superior conductivity for potassium ions (Fig. S15). This property has a positive impact on critical performance metrics such as power density and energy efficiency of the aqueous organic flow battery.

**Fig. 4:**
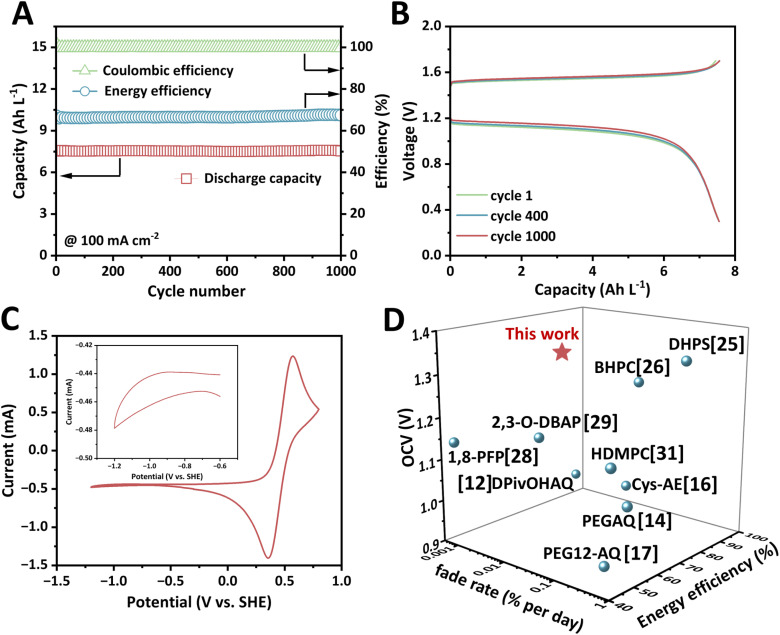
Long-cycling performance of K_4_Fe(CN)_6_‖dMeODBAP AOFB-blend. The negolyte is 6 mL of 0.2 M dMeODBAP in 1 M KOH solution and the posolyte is 80 mL of 0.1 M K_4_Fe(CN)_6_ and 0.025 M K_3_Fe(CN)_6_ in 1 M KOH solution. (A) Long-term cycling performance of K_4_Fe(CN)_6_‖dMeODBAP AOFB-blend at a current density of 100 mA cm^−2^. (B) Charge–discharge voltage profile of dMeODBAP from selected cycles. (C) CV image of the posolyte from the 0.2 M dMeODBAP‖K_4_Fe(CN)_6_ cell after the cycling test without being diluted. The inset image is magnified from −0.6 V to −1.2 V. (D) Battery performances of the dMeODBAP-based AOFB compared with those of previously reported alkaline AOFBs.

The exceptional stability of dMeODBAP prompts us to investigate the long-term cycling performance of the dMeODBAP‖K_4_Fe(CN)_6_ full cell. Fig. S16 showcases the open circuit voltages (OCVs) of AOFB-blend measured at various SOCs, which increase linearly from 12.5% to 100% SOC. The OCV at 50% SOC is 1.341 V, which is in line with the CV results in Fig. 2A. Impressively, the OCV is measured to be 1.39 V at around 100% SOC, which is a considerably competitive value among most reported alkaline AOFBs (Table S3). The long-term cycling performance of dMeODBAP‖K_4_Fe(CN)_6_ AOFB-blend is shown in Fig. 4A. When the battery is operated at 100 mA cm^−2^ for 1000 cycles, energy efficiency remains at 66–67% steadily, and coulombic efficiency also remains above 99.8%. The discharge capacity fades slightly from 7.555 to 7.551 A h L^−1^, corresponding to a low fade rate of 0.007% per day and a remarkable capacity retention of 99.95%. And during the long cycling test the whole cell delivers an average energy density of 7.86 W h L^−1^ based on the negolyte. The charge and discharge curves at various cycles are compared and they are almost the same, demonstrating the outstanding stability of AOFB-blend (Fig. 4B).

The long cycling stability of AOFB-N212 is also investigated at 100 mA cm^−2^ for 1000 cycles (Fig. S17). As depicted in Fig. S17A, the discharge capacity remains stable at around 6.1 A h L^−1^ during the first 400 cycles and no apparent capacity fade is observed. The energy efficiency is also stable at around 61.3%. However, capacity decay has taken place since the 400th cycle, gradually decreasing from 6.16 to 5.76 A h L^−1^, and the energy efficiency also declined from 61.4 to 59.6% (Fig. S17B and C). The capacity utilization declined due to the upward and downward shift of the charge and discharge curves, respectively. The CV image of the posolyte in AOFB-blend after cycling presents no recognizable signal of dMeODBAP, indicating that capacity decay caused by permeation is negligible (Fig. 4C). The electrochemical performances of dMeODBAP‖K_4_Fe(CN)_6_ AOFB-blend are compared with those of previously reported alkaline AOFBs in Fig. 4D, indicating its high voltage, exceptional cycling stability and high energy efficiency at 100 mA cm^−2^.

In order to investigate the mechanism of performance degradation of AOFB-N212, we retrieved the membranes from the AOFBs when the long cycling test was completed. As shown in Fig. S18, the blend membrane basically maintained its original colour with no mechanical failure observed. However, the Nafion 212 membrane changed from its original colourless and transparent state to a dark brown colour. These contaminants could not be removed by rinsing with deionized water or ultrasonic treatment. We suppose that some dMeODBAP was adsorbed on the surface of the Nafion 212, blocking ion transport pathways and leading to an increase in membrane resistance. The increased resistance leads to a gradual decline in capacity utilization and energy efficiency. Moreover, the adsorbed dMeODBAP hardly returned to the solution phase, resulting in a loss of capacity in another way. In other words, AOFB-N212 demonstrated good stability during the initial 400 cycles due to the uncontaminated Nafion 212 and the stable dMeODBAP. After that, the contamination of the membrane got worse and led to an increased resistance, which degraded the battery's capacity utilization and energy efficiency. The blend membrane was not seriously contaminated because of its relatively small pore size. Also, dMeODBAP itself has a bulky molecular size, experiencing a strong size-exclusion effect from the blend membrane. In addition, in order to evaluate the practical application potential of dMeODBAP, its production cost is roughly estimated according to lab-scale manufacturing (Table S4). It approximately costs $1.64 to synthesize one gram and the prices of it and its analogues are expected to be even lower if the synthesis routes and conditions are further optimized.

## Conclusions

In conclusion, we report a new type of low-redox-potential phenazine-based molecule, dMeODBAP. Since four electron-donating substituents are introduced into the phenazine core, its redox potential is as low as −0.84 V (*vs.* SHE). Meanwhile, four substituents endow dMeODBAP with a relatively bulky molecular size, which effectively mitigates the capacity decay caused by crossover. In addition, characterizations confirm the high redox reversibility and exceptional chemical stability of dMeODBAP. Furthermore, a new microporous blend membrane is prepared and exhibits a higher potassium ion conductivity than the commercial Nafion 212 membrane. Therefore, benefiting from the low-potential dMeODBAP and the blend membrane, the full cell achieves a high voltage of 1.34 V and demonstrates a high energy efficiency of approximately 67% and exceptional capacity retention of approximately 99.95% for 1000 cycles at 100 mA cm^−2^, corresponding to a low fade rate of 0.007% per day. The AOFB based on Nafion 212 shows a higher capacity decay, which is attributed to the increasing resistance and performance degradation caused by the adsorption of dMeODBAP on the membrane.

## Author contributions

Y. Wang and X. Xie conceived the idea and designed the experiments. X. Xie and T. Kong carried out the materials synthesis, characterization and cell testing. J. Gao and R. Li discussed the mechanism. X. Xie and Y. Wang wrote the draft. Y. Wang supervised the project. All authors discussed the results and commented on the manuscript.

## Conflicts of interest

There are no conflicts to declare.

## Supplementary Material

SC-OLF-D5SC07193C-s001

## Data Availability

The data supporting this article have been included as part of the supplementary information (SI). Supplementary Information: experimental section and additional figures and tables. It contains NMR spectra, results of theoretical calculation, UV-vis absorption spectra, CV curves, EIS curves, galvanostatic charge–discharge voltage profiles, results of permeability measurements, battery performance, the photos of membranes, battery voltage comparsion and cost calculation. See DOI: https://doi.org/10.1039/d5sc07193c.
