# Supplementary material for: A low-redox-potential phenazine-based negolyte with high stability for aqueous organic flow batteries
Source: Chem Sci. 2025 Oct 23;16(47):22368–75. doi: 10.1039/d5sc07193c (PMC12560832; doi:10.1039/d5sc07193c)
Supplement: SC-016-D5SC07193C-s001 [file SC-016-D5SC07193C-s001.pdf]

Supplementary Information (SI) for Chemical Science.
This journal is © The Royal Society of Chemistry 2025

Supplementary Information

A Low-Redox-Potential Phenazine-based Negolyte with High Stability for Aqueous Organic Flow Batteries

*Xuanyu Xie[1], Taoyi Kong[1], Jiaming Gao[1], Ruiyang Li[1], and Yonggang Wang[1]**

1. Department of Chemistry and Shanghai Key Laboratory of Molecular Catalysis and Innovative Materials, Institute of New Energy, iChEM (Collaborative Innovation Center of Chemistry for Energy Materials), Fudan University, Shanghai 200433, China

E-mail: ygwang@fudan.edu.cn

Experimental Section

General information for materials

Unless otherwise specified, all chemicals were used directly without further purification. Nitric acid, hydrochloric acid, KOH, ethanol, hydrazine hydrate (85 %), potassium tert-butoxide, anhydrous K_2CO_3, 2-propanol, dimethyl sulfoxide, sulfuric acid and glacial acetic acid were purchased from Sinopharm Chemical Reagent Co. (China). 2,5-dihydroxy-1,4-benzoquinone, tetrafluoroterephthalonitrile (TFTPN) and 10 % Pd/C powder were purchased from Bide Pharmatech Ltd. (China). 1,2-dimethoxybenzene, N,N-dimethylforamide, methyl 4-bromobutyrate, 5,5',6,6'-Tetrahydroxy-3,3,3',3'-tetramethyl-1,1'-spirobiindane (TTSBI) and 9,9-bis(3,4-dihydroxyphenyl)fluorene (BDPF) were purchase from Shanghai Aladdin Bio-Chem Technology Co., Ltd. Nafion 212 membranes were purchased from DuPont Corp., USA. TTSBI was purified according to a previous study[1]. Unless stated otherwise, all reactions were carried out under the protection of argon gas.

Synthetic Procedures of polymers

A mixture of TFTPN (0.512 g, 2.56 mmol) and TTSBI (0.872 g, 2.56 mmol) were stirred in 13 mL of dry DMF until the monomers dissolved. Then anhydrous K_2CO_3 (1.06 g, 7.68 mmol) and additional DMF (4.3 mL) were added to the solution (denoted as solution A). Meanwhile, another mixture of TFTPN (0.512 g, 2.56 mmol) and BDPF (0.98 g, 2.56 mmol) were also stirred in a same volume of DMF following the addition of K_2CO_3 and DMF (denoted as solution B). Solution A and B were stirred at 65 °C for 24 hours and then solution A was transferred into the solution B carefully. The mixture was then stirred at 65 °C for 48 hours. Finally, the mixture was added to water (100 mL) after cooling and the crude product collected by filtration. Repeated reprecipitations from methanol gave fluorescent yellow polymer TTB-PIM-1 (2.02 g, 85 %). To further eliminate low-molecular-weight impurities intermingled in the crude product, Soxhlet extraction with methanol was performed for 24 hours. TTB-PIM-COOH was synthesized via a facile acid hydrolysis method according to previous reports[2, 3]. TTB-PIM-1 (1 g), deionized water (60 mL), glacial acetic acid (20 mL) and sulfuric acid (60 mL) were sequentially added and stirred at 300 rpm at 155 °C for 48 hours. On cooling, the heterogeneous solution was neutralized with 200 mL of deionized water for three times and the brown-yellow powder was filtered. To remove residual reagents, the powder was refluxed in a 0.5 M sulfuric acid aqueous solution (140 mL) for approximately 12 hours, filtered, and vacuum-dried at 120 °C overnight (0.97 g, 90 %). AO-PIM-1 was prepared following the procedure reported previously[4].

The preparation of the blend membrane

Stirred at 60 °C, TTB-PIM-COOH powder (0.2 g), AO-PIM-1 powder (0.2 g) and polybenzimidazole (0.2 g) were dissolved in a mixed solvent (9.4 g, DMSO & DMF, 5:5 ratio) to form a casting solution. The solution was centrifuged at 9000 rpm for 15 minutes to remove insoluble impurities, then cast onto a clean glass plate and allowed to flow naturally. The membrane was formed through slow evaporation of solvent in an oven at 60 °C for 8 hours. Subsequently, it was further dried under vacuum for overnight to remove residual solvent. The dry membrane (a thickness of 60 μm approximately) was peeled off from the glass plate and immersed into 1 M KOH at 80 °C for 8 hours ahead of battery testing.

Synthetic Procedure of dMeODBAP

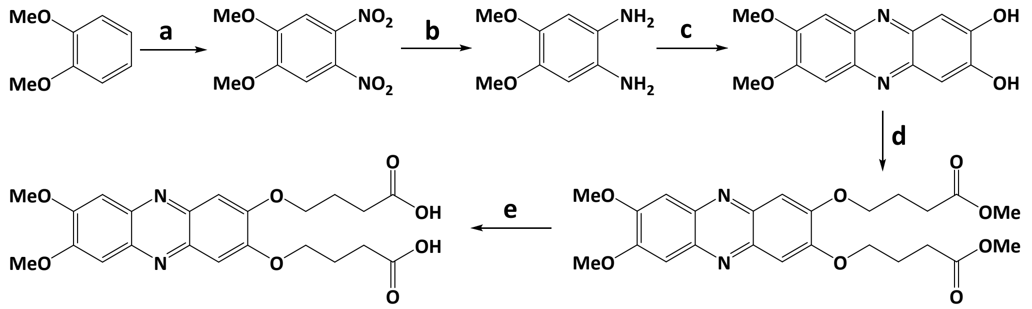

The following presents the standard synthesis procedure and the amounts of the materials should be adjusted according to the actual requirements. **(a)** Under an ice bath, 5 mL of 1,2-dimethoxybenzene was added to 33 mL of nitric acid in five portions, and the mixture was vigorously stirred and refluxed at 80 °C for 5 hours. Upon cooling the reaction mixture was subjected to filtration. The residue was washed with deionized water repeatedly until the filtrate reached a neutral pH. Subsequently, the light-yellow solid was vacuum-dried at 60 °C overnight to obtain 1,2-dimethoxy-4,5-dinitrobenzene. **(b)** 4.26 mL of hydrazine hydrate (85 %) was slowly added into the mixture of 10 % Pd/C catalyst (0.5 g), 1,2-dimethoxy-4,5-dinitrobenzene (2 g) and ethanol (40 mL) and then refluxed at 80 °C for 2.5 hours. After cooling to room temperature, the mixture was filtered and the residue was washed with 30 mL of ethanol in three portions. The filtrate was collected and quickly subjected to rotary evaporation. Finally, the white-off solid was vacuum-dried at 60 °C overnight to obtain 4,5-dimethoxybenzene-1,2-diamine. It should be particularly noted that this product is extremely prone to be oxidated, which leads to the presence of oxidation impurities in the actual product, exhibiting a light-yellow colour. **(c)** 4,5-dimethoxybenzene-1,2-diamine (1.24 g) and 2,5-dihydroxy-1,4-benzoquinone (1.03 g) were added into 80 mL water and refluxed for 7 hours. Upon cooling

the mixture was filtered and the residue was washed with deionized water. The greenish solid was vacuum-dried at 80 °C overnight to obtain 7,8-dimethoxyphenazine-2,3-diol. **(d)** 1.04 g potassium *tert*-butoxide, 0.73 g 7,8-dimethoxyphenazine-2,3-diol and 34 mL DMF were mixed and stirred at room temperature for 15 minutes. Then 1.84 g anhydrous potassium carbonate and 1.59 mL methyl 4-bromobutyrate were added into the solution. The mixture was stirred at 95 °C overnight. Upon cooling the mixture was poured into 20 mL ice water and filtered. The dark brown solid was washed with 0.1 M KOH solution and deionized water until the filtrate was colourless, and then vacuum-dried at 80 °C overnight. **(e)** This product (0.91 g) was added into 40 mL deionized water and 20 mL 2-propanol with 0.534 g potassium hydroxide. The mixture was stirred at 60 °C for half an hour and filtered after cooled down. The filtrate was diluted with 200 mL of water and the pH was adjusted to around 5 by hydrochloric acid. The suspension was subjected to rotary evaporation and the obtained dark brown solid was washed with deionized water. The final product dMeODBAP was obtained after vacuum-dried at 80 °C overnight.

Materials characterization

^{1}H nuclear magnetic resonance (NMR) measurements were run with a 400 MHz liquid nuclear magnetic resonance spectrometer (AVANCE III HD, Bruker) and 10 mg of the compounds was dissolved in 0.6 mL DMSO-d_6 and filtered before test. Ultraviolet-visible spectroscopy (UV-Vis) were measured using a PerkinElmer Lambda 35 spectrometer at a wavelength interval of 0.1 nm.

Ionic conductivity measurement

Ionic conductivity of the blend membrane and Nafion 212 was measured on electrochemical impedance spectroscopy (EIS) using the potentiostatic mode at an AC bias of 10 mV and a frequency range from 1 MHz to 10 Hz. For apparent ionic conductivity tests, membrane samples were pretreated in 1 M aqueous KOH overnight to fully deprotonate hydroxyl groups, followed by soaking in deionized H_2O for 24 hours to remove residual KOH, then equilibrated in 1 M KOH aqueous electrolyte for 24 h. Membrane samples were sandwiched between two stainless steel electrodes and sealed with coin cells (Type 2032). The ionic conductivity in the range of 30 to 80 °C was calculated according to:

$$\sigma = \frac{L}{A \times R}$$

where σ is the membrane ionic conductivity (S cm^{-1}), L is the membrane thickness (cm), A is the membrane active area of 2.83 cm^2, and R is the membrane resistance (Ω). The resistance of blank coin cell (0.04 Ω) was subtracted from R before applying the Equation above.

Permeability measurements

Redox-active molecule permeability tests were performed using concentration-driven dialysis diffusion H-shaped cells. The blend membrane and Nafion 212 were sandwiched between two chemically resistant O-rings and secured in the middle of H-cells by clips respectively. Continuous stirring was used in both donating and receiving sides to alleviate the concentration polarization near the membrane. The donating side comprised of 0.2 M dMeODBAP in 20 mL 1.2 M KOH. The receiving side comprised of 0.2 M K_2SO_4 in 20 mL 1 M KOH. At different time intervals, 2 mL solution was taken from the receiving side and characterized by UV-vis spectroscopy. Upon completion of testing, the solution was returned to the receiving side. The concentration of dMeODBAP in the receiving side was calculated according to pre-calibrated absorbance-concentration curves and the permeability (P) was calculated according to Fick equation:

$$P = \frac{V_0 l}{2A} \times \frac{\Delta ln(1 - \frac{2C_t}{C_0})}{\Delta t}$$

with following parameters: V_0 = 20 mL, A = 2.01 cm^2, l = 50 μm. C_0 is the original concentration of dMeODBAP in donating side, V_0 is the solution volume in both sides, l is the membrane thickness and A is the membrane area.

Cyclic Voltammetry (CV) Measurement

CV measurements were conducted with an Autolab potentiostat (PGSTAT302N). A glassy carbon working electrode (0.15 cm radius), a Hg/HgO reference electrode (0.098 V vs. SHE) and a spiral platinum counter electrode (0.5*150 mm) made up the three-electrode system. The working electrode was polished with Al_2O_3 powder (50 nm) and rinsed with deionized water before test. CV curves were acquired using 5 mM dMeODBAP in 1 M KOH solution. The CV curves at various pH values ranging from 14.3 to 7 were recorded using a pH meter (Leici PHS-3C) and an Ag/AgCl reference electrode (0.197 V vs. SHE).

Solubility measurement

111 mg dMeODBAP (0.25 mmol) was mixed with 0.5 mmol KOH in a centrifuge tube. Then 1 M KOH solution was added drop by drop into the tube under ultrasonicated until few solid

remained visible. The aliquot was filtered and diluted by 10 mM CH_3SO_3Na in D_2O with a ratio of 1:5 before characterized by ^{1}H-NMR. The concentration was calculated according to following formula:

$$S = 0.025 \times Area_{3.41\,ppm} \div Area_{2.68\,ppm}$$

DFT calculations

For all calculations, the B3LYP functional is adopted with the D3BJ dispersion correction. In the calculations of geometry optimization and frequency, basis set of 6-31+G (d, p) was adopted for all atoms. The single point energy was calculated under the level of M062X-D3/6-311+G (d, p). When conducting optimization and frequency calculations, the Solvation Model Based on Density (SMD) implicit solvation model was chosen for the consideration of solvation effect. Water was selected as the solvent.

Linear sweep voltammetry (LSV) with rotating disk electrode (RDE)

LSV measurements were carried out with an Autolab potentiostat (PGSTAT302N). A rotating glassy carbon working disk electrode (0.25 cm radius), a Hg/HgO reference electrode (0.098 V vs. SHE) and a spiral platinum counter electrode (0.5*150 mm) made up the three-electrode system. The working electrode was polished with Al_2O_3 powder (50 nm) and rinsed with deionized water before test. The working electrode was rotated at a specific speed while the applied potential was swept at a scan rate of 10 mV s^{-1}. The diffusion coefficients (D) of dMeODBAP were calculated according to Levich equation:

$$i_{limit} = 0.62nFAD^{2/3}\omega^{1/2}v^{-1/6}C$$

where n = 2, F = 96485 C mol^{-1}, A = 0.1963 cm^2, C = 5×10^{-6} mol cm^{-3}, v = 0.0096 cm^2 s^{-1}. The Koutecky-Levich plots at different overpotentials were extrapolated to get the kinetic current (i_k) according to the Koutecky-Levich equation:

$$\frac{1}{i} = \frac{1}{i_k} + \frac{1}{0.62nFAD^{2/3}\omega^{1/2}v^{-1/6}C}$$

the reaction rate constant (k_0) and transfer coefficient (α) were determined according to Butler-Volmer equation:

$$\log i_k = \log nFACk_0 - \frac{\alpha nF}{2.303RT}\eta$$

Cell assembly

Full cell tests were carried out on a Landt batteries tester. Two pieces of graphite plates with serpentine flow patterns (Beijing Jinglong Special Carbon Technology Co., Ltd.) were used as

the negative electrode and positive electrode respectively. Both electrodes were covered by 4 pieces of WOS1011 carbon cloth pre-heat in air at 400 °C for 24 hours. Nafion 212 or the blend membrane was used as the ion-exchange membrane. Before tests, the membranes were immersed into 1 M KOH solution at 80 °C for 8 hours. The electrolyte was circulated between the cell stack and the tank through peristaltic pumps (Runze Fluid) at a rate of around 60 mL min^{-1}. The composition of posolyte and negolyte were various in different tests. In the SOC-ASR-power density and long-term stability test, the cell comprised of 6 mL 0.2 M dMeODBAP in 1.4 M KOH as the negolyte, 80 mL 0.1 M $K_4Fe(CN)_6$ and 0.025 M $K_3Fe(CN)_6$ in 1 M KOH as the posolyte. In the in-situ UV-vis test, the cell comprised of 25 mL 8 mM dMeODBAP in 1 M KOH as the negolyte, 50 mL 8 mM $K_4Fe(CN)_6$ in 1 M KOH as the posolyte.

In-situ UV-vis spectroscopic measurement

In the in-situ UV-vis measurement of the dMeODBAP solution (its concentration is 8 mM), a 100 μm optical path-length flow quartz cell was linked to the flow battery. 1 M KOH solution was used as the blank sample. The battery was charged to 1.6 V at 50 mA (10 mA cm^{-2}) and then charged at 1.6 V until the current was smaller than 20 mA (4 mA cm^{-2}). After that, the cell was discharged to 0 V at 50 mA (10 mA cm^{-2}). The wavelength range of the UV spectrum is 700 nm to 200 nm, and the scanning speed is 1920 nm min^{-1}.

Price calculation

The lab-scale reagent grade prices were evaluated based on the following equation:

$$U_{dMeODBAP} = \frac{\sum_{reactant} EUM}{Y \times M_{dMeODBAP}}$$

where U represents the cost (\$ g^{-1}), E represents the reaction equivalent, M represent the molar weight (g mol^{-1}) and Y represents the yield (approximately 55 % overall yield for dMeODBAP).

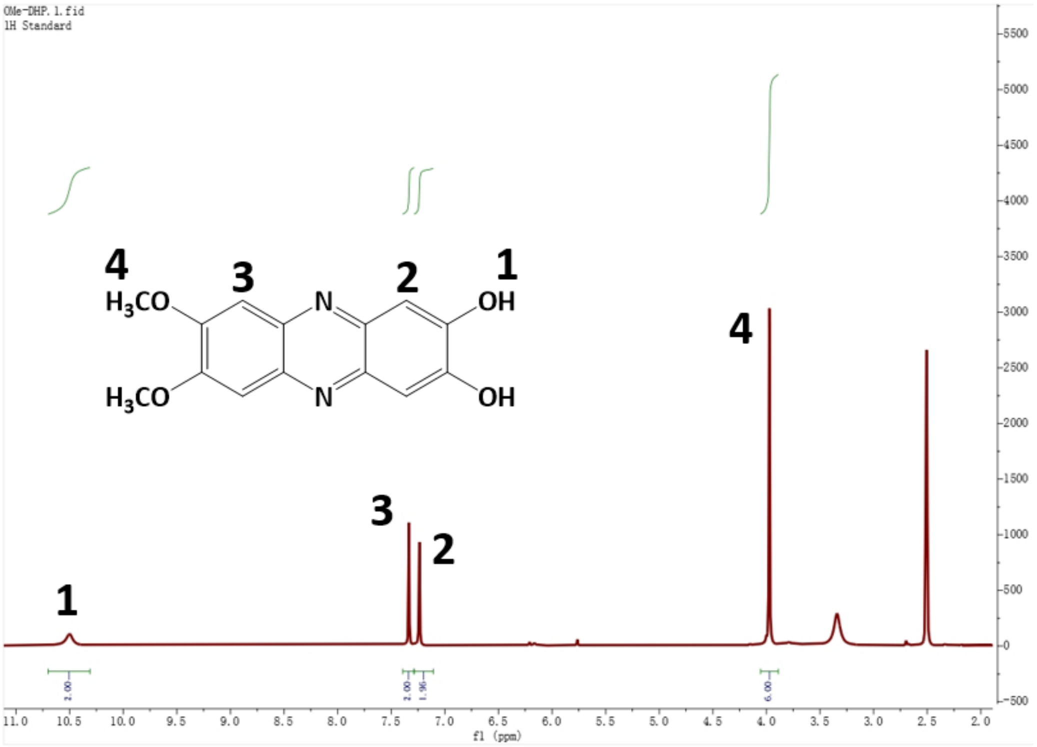

Supplementary Figure. 1. ^1H NMR spectra of dMeO-DHP. Peak at 2.5 ppm is from DMSO-d_6, and peak at 3.33 ppm is from H_2O.

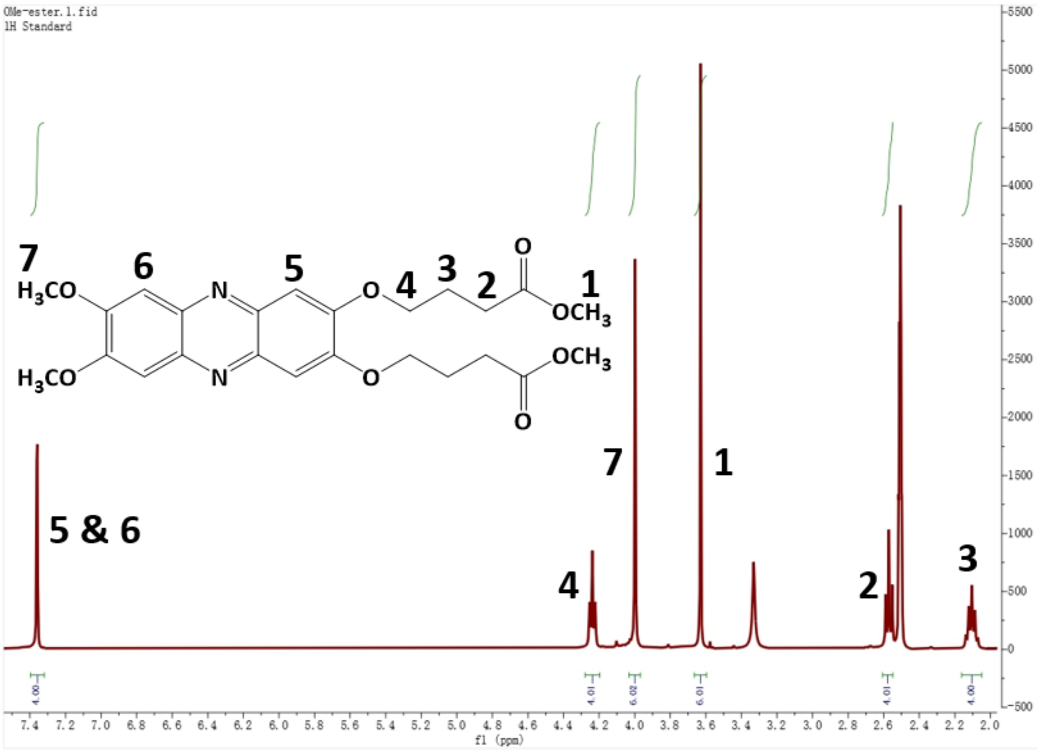

Supplementary Figure. 2. ^{1}H NMR spectra of dMeO-ester. Peak at 2.5 ppm is from DMSO-d_6, and peak at 3.33 ppm is from H_2O.

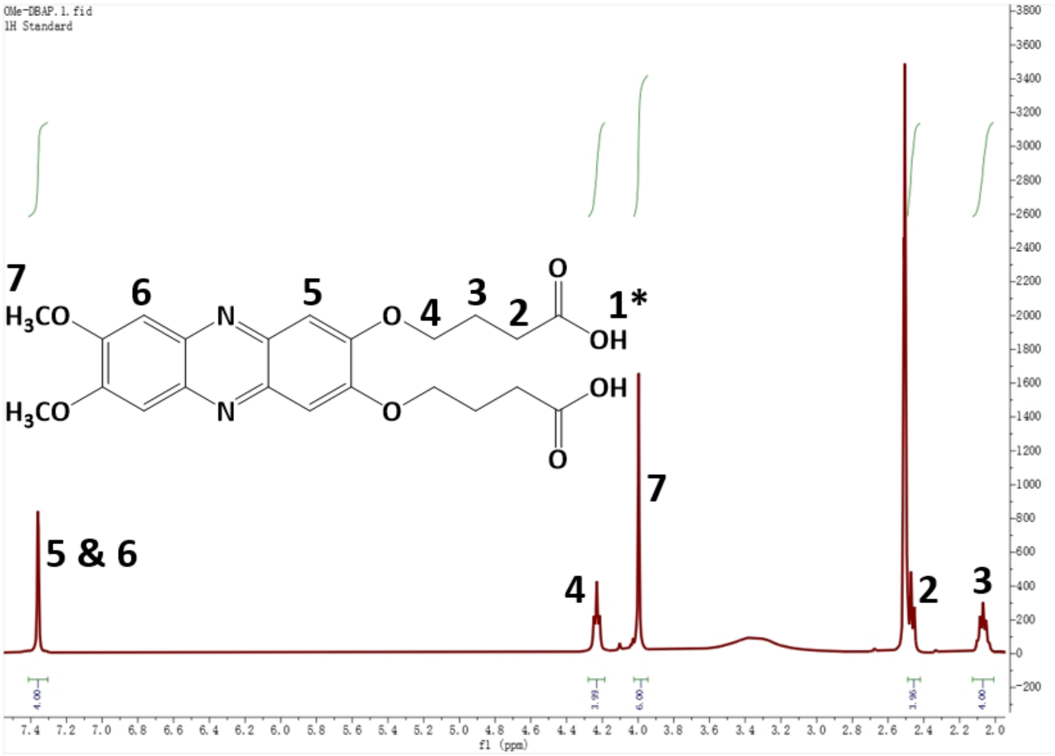

Supplementary Figure. 3. ^{1}H NMR spectra of dMeODBAP. Peak at 2.5 ppm is from DMSO-d_6, and peak at 3.33 ppm is from H_2O.

*The carboxyl hydrogens did not yield detectable signal peak.

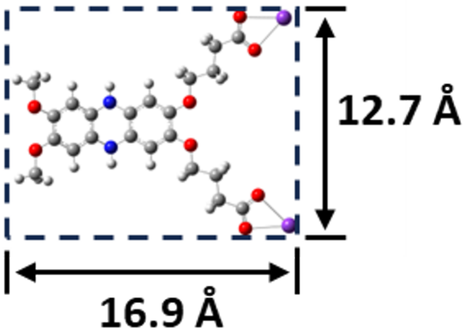

Supplementary Figure. 4. The external contour of dMeODBAP is simplified to a rectangle and the lengths of its short and long sides.

Gray spheres represent carbon atoms; white spheres represent hydrogen atoms; red spheres represent oxygen atoms; blue spheres represent nitrogen atoms; and purple spheres represent potassium ions.

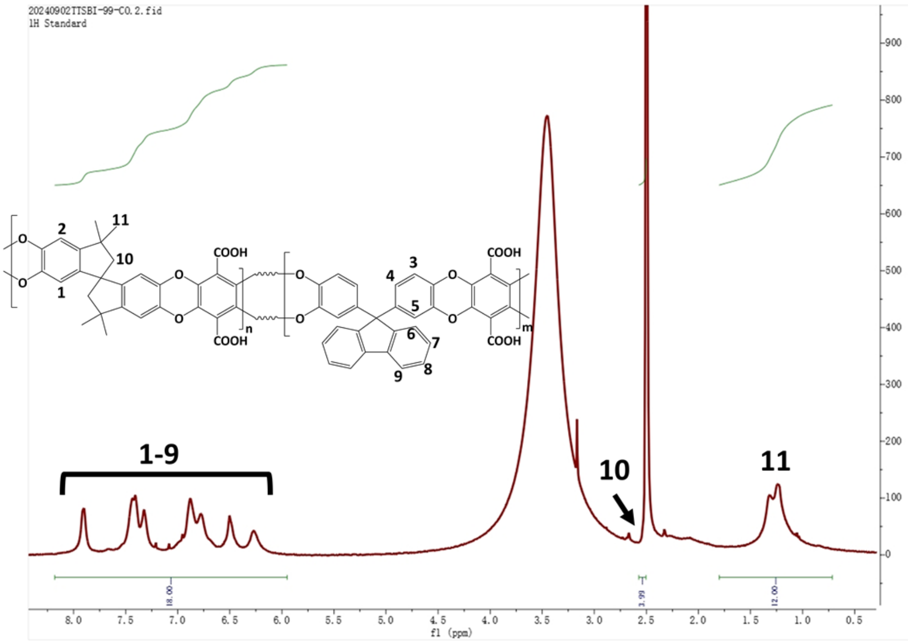

Supplementary Figure. 5. ^{1}H NMR spectrum of TTB-PIM-COOH. ^{1}H NMR (400 MHz DMSO-d_6) spectrum of TTB-PIM-COOH. Peak at 2.5 ppm is from DMSO-d_6, and peak at 3.33 ppm is from H_2O.

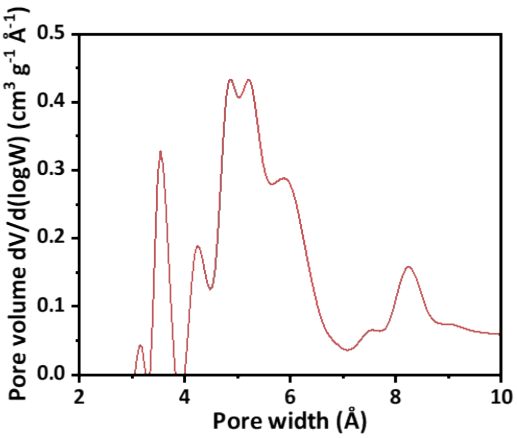

Supplementary Figure. 6. Pore size distribution derived from CO_2 sorption isotherm according to density-functional theory (DFT) calculations.

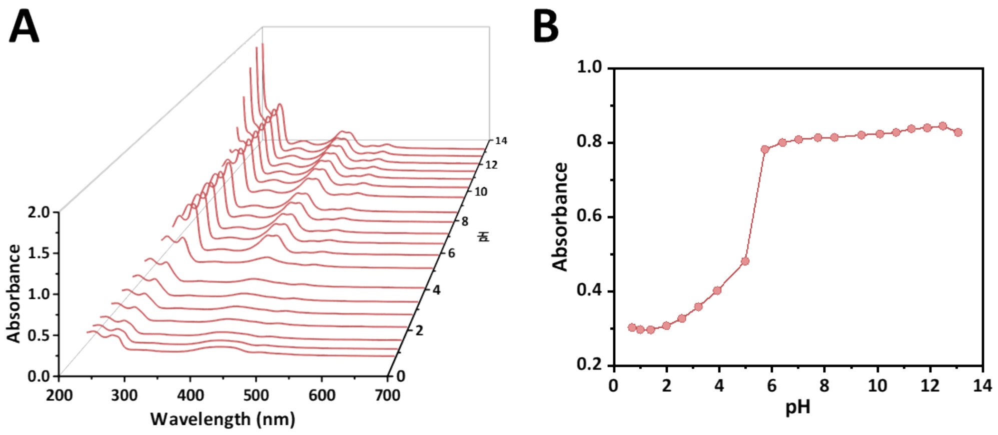

Supplementary Figure. 7. (A) UV–vis absorption spectra of dMeODBAP solution at different pH values. **(B)** Light absorbance vs. pH value plots of the dMeODBAP solutions at $\lambda_{254\ nm}$.

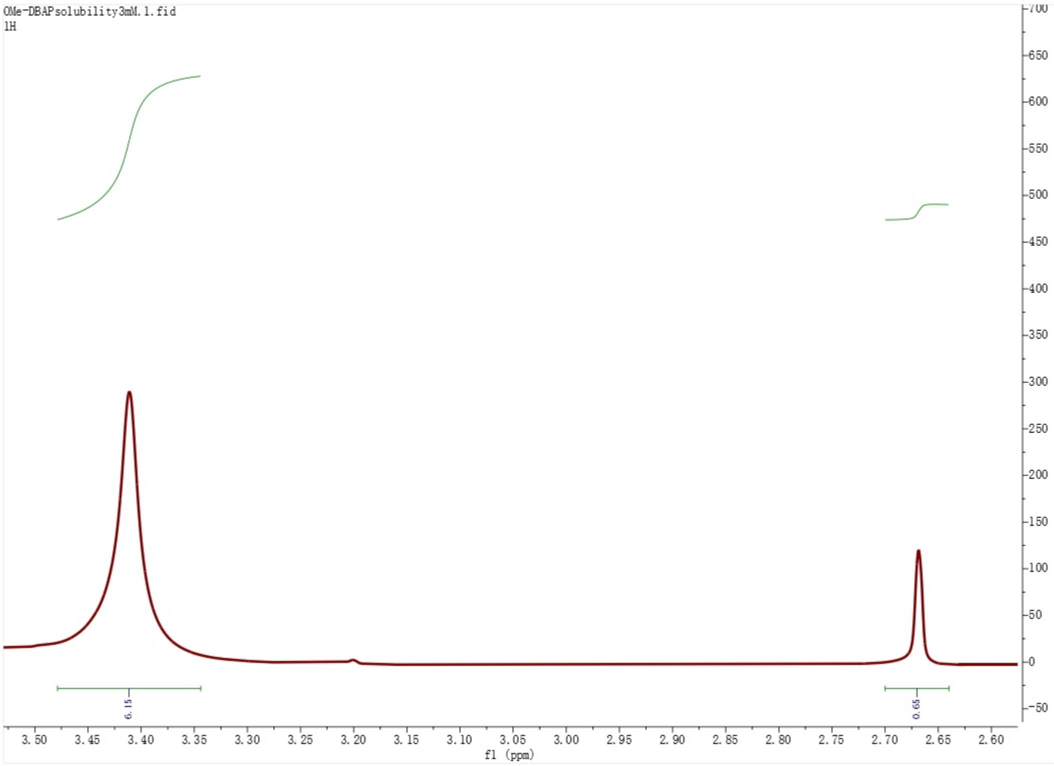

Supplementary Figure. 8. The measurement of the solubility of dMeODBAP by ^{1}H NMR. Peak at 2.68 ppm is from CH_3SO_3Na and peak at 3.41 ppm is from methoxy groups in dMeODBAP.

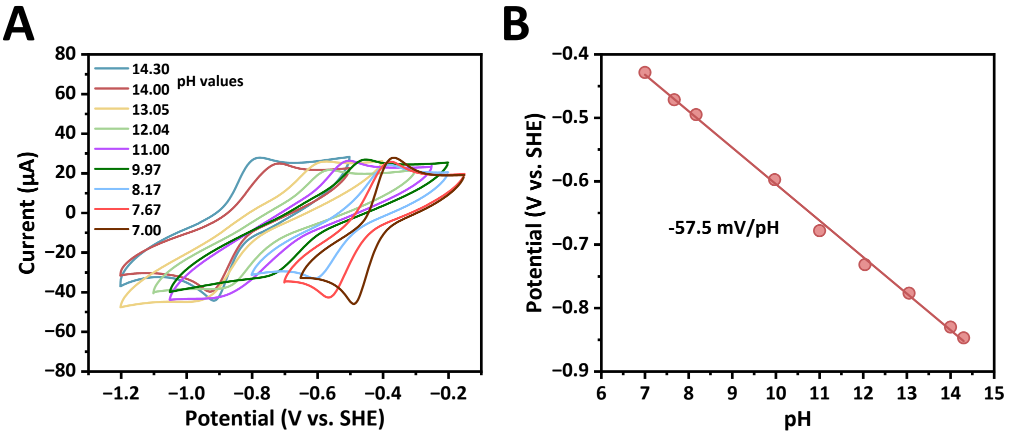

Supplementary Figure. 9. (A) CV curves of dMeODBAP solution at different pH values with a scan rate of 25 mV s^{-1}. **(B)** Pourbaix diagrams of dMeODBAP.

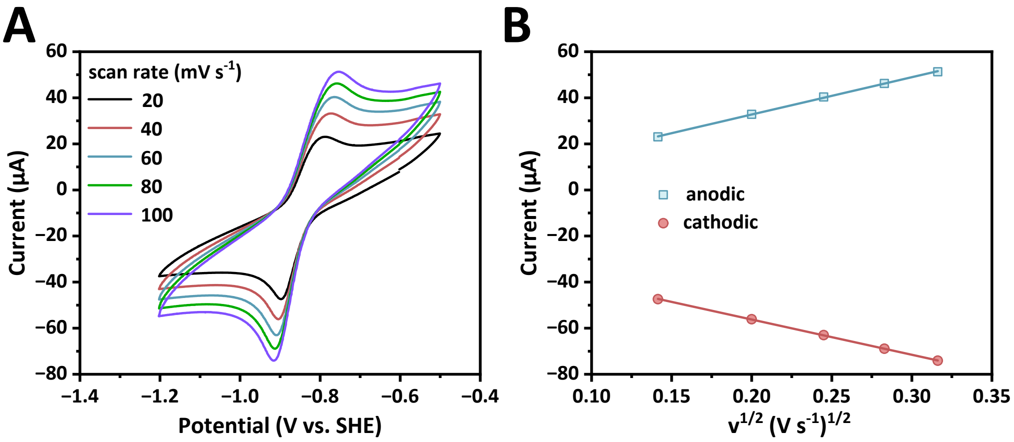

Supplementary Figure. 10. (A) CV images of 5 mM dMeODBAP in 1 M KOH at varying scan rates. **(B)** Peak currents vs square roots of scan rates of dMeODBAP.

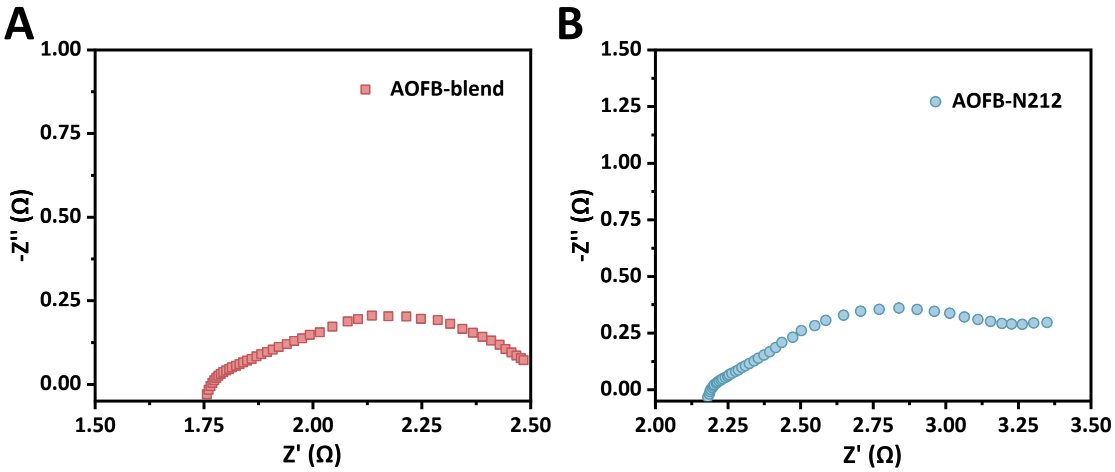

Supplementary Figure. 11. Area-specific resistance (ASR) of (A) AOFB-blend and (B) AOFB-N212. The effective area is 1 cm^{-2}.

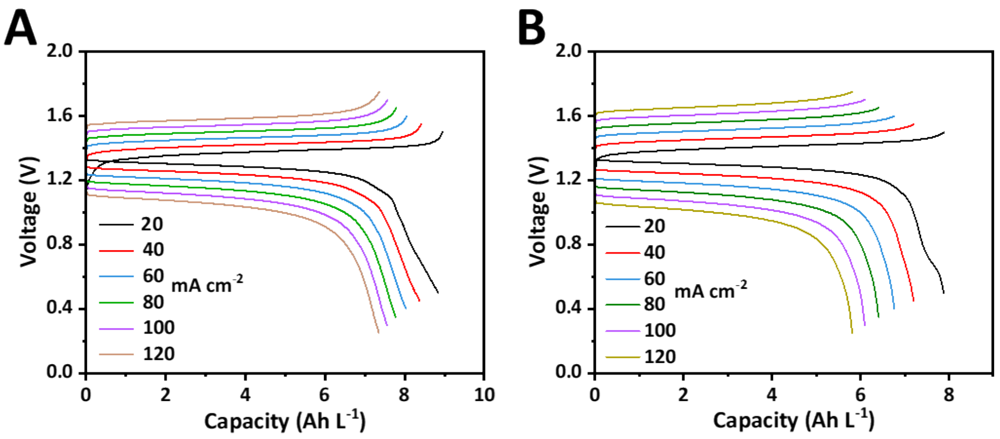

Supplementary Figure. 12. (A) Galvanostatic charge-discharge voltage profiles of AOFB-blend from 20 mA cm^{-2} to 120 mA cm^{-2}. **(B)** Galvanostatic charge-discharge voltage profiles of AOFB-N212 from 20 mA cm^{-2} to 120 mA cm^{-2}.

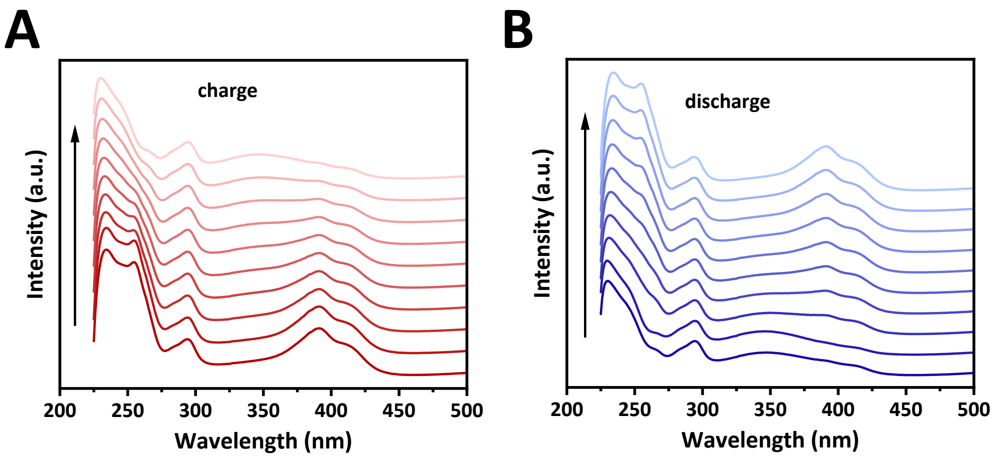

Supplementary Figure. 13. (A) In situ UV-vis absorption spectra of dMeODBAP during charging process. **(B)** In situ UV-vis absorption spectra of dMeODBAP during discharging process.

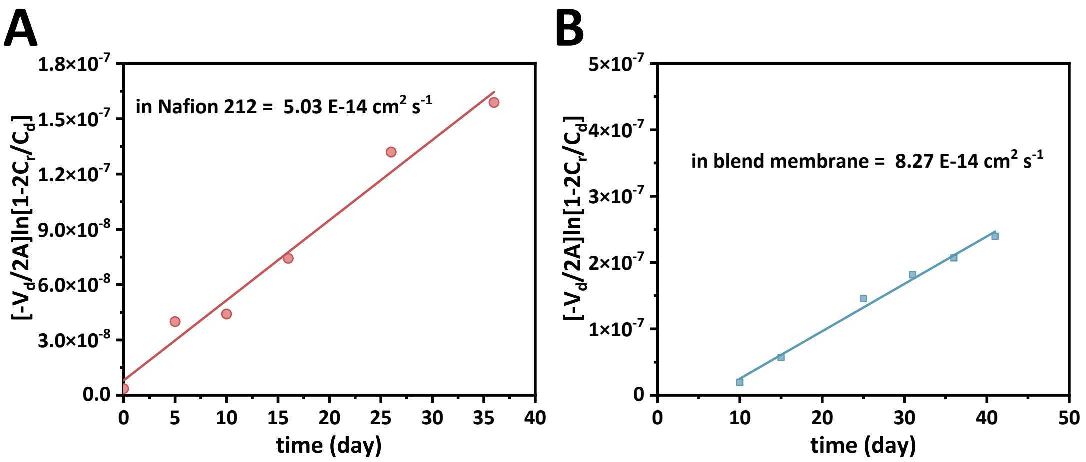

Supplementary Figure. 14. (A) The permeability of Nafion 212 membrane for dMeODBAP solution. **(B)** The permeability of the blend membrane for dMeODBAP solution.

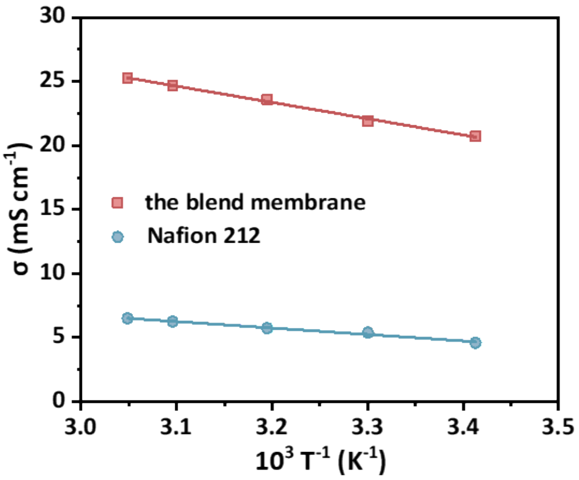

Supplementary Figure. 15. Ionic conductivity of Nafion 212 and the blend membrane measured in 1 M KOH solution.

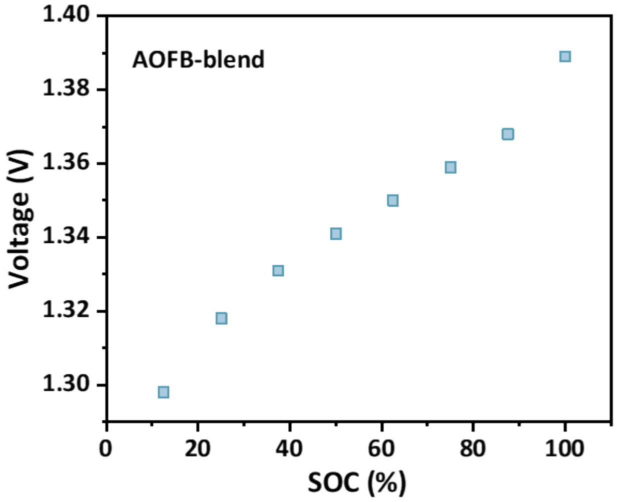

Supplementary Figure. 16. OCV vs. SOC curve of the AOFB-blend.

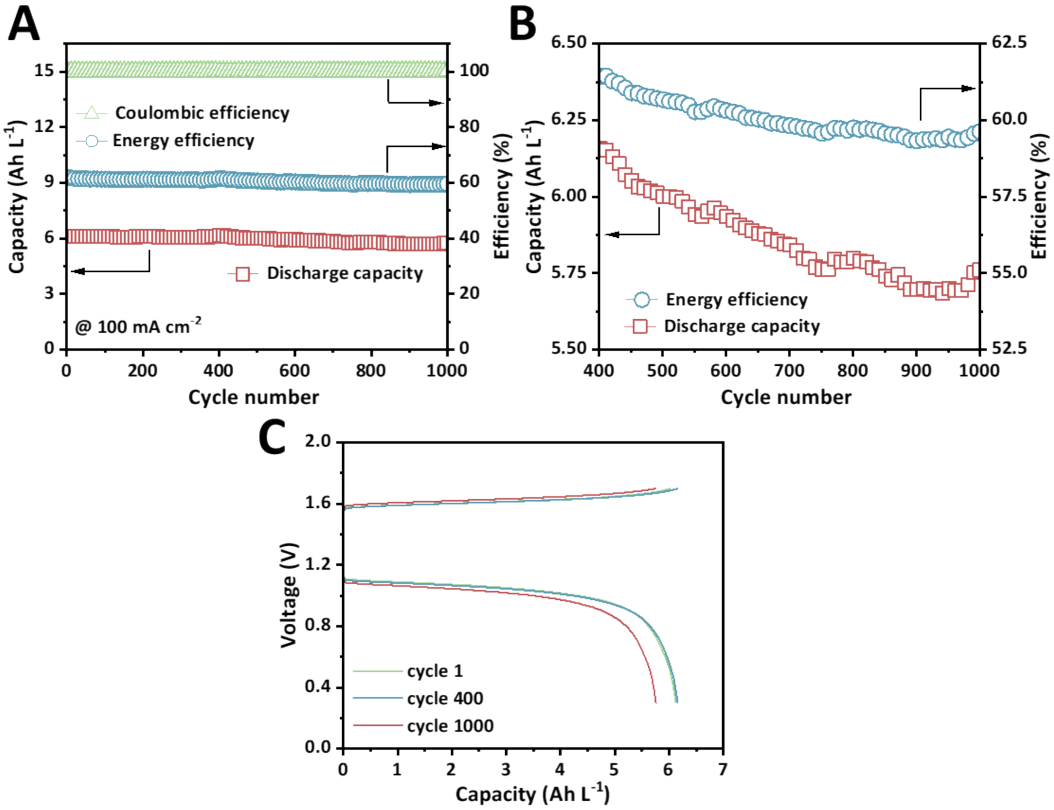

Supplementary Figure. 17. (A) Long-term cycling performance of dMeODBAP||$K_4Fe(CN)_6$ AOFB-N212 at a current density of 100 mA cm^{-2}. **(B)** The discharge capacity and energy efficiency of AOFB-N212 from 400th to 1000th cycle. **(C)** Charge-discharge voltage profile of AOFB-N212 from selected cycles in **Supplementary Figure 16A**.

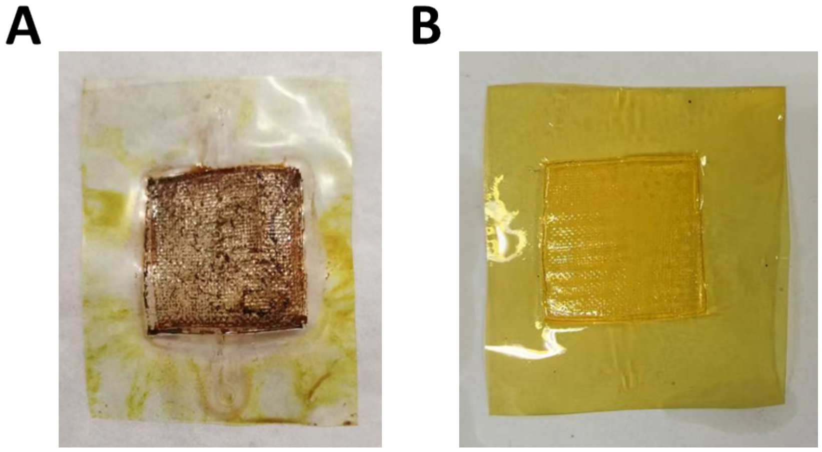

Supplementary Figure. 18. (A) The cycled Nafion 212 membrane disassembled from dMeODBAP||$K_4Fe(CN)_6$ full cell. **(B)** The cycled blend membrane disassembled from dMeODBAP||$K_4Fe(CN)_6$ full cell. Both of their effective areas are 5 cm^2.

Supplementary Table 1. Redox potential of dMeODBAP and previous reported negolytes in alkaline AOFBs.

Redox-active molecules	Redox potential at pH 14 (V vs. SHE)
**dMeODBAP (this work)**	**-0.84**
2,6-DHAQ[5]	-0.7
2,6-DBEAQ[6]	-0.515
2,6-DPPEAQ[7]	-0.47
DAEAQ[8]	-0.61
AQ-1,8-3E-OH[9]	-0.52
PEG12-AQ[10]	-0.443 (neutral)
DCDHAQ[11]	-0.56
DPivOHAQ[12]	-0.48
2,6-D2PEAQ[13]	-0.445
AQDP[14]	-0.456
Cys-AE[15]	-0.51
Cys-DHAQ[16]	-0.492
DHPS[17]	-0.86
BHPC[18]	-0.78
1,6-DPAP[19]	-0.56 (pH 12)
1,8-PFP[20]	-0.588
2,3-O-DBAP[21] (DBEP[22])	-0.699 (-0.68)
HDMPC[23]	-0.722
HSPC[24]	-0.42
4C7SFL[25]	-0.7
ds-BC[26]	-0.64

Supplementary Table 2. Optimized coordinates and energies of re-dMeODBAP and the two tr-dMeODBAP isomers.

re-dMeODBAP

Zero-point correction = 0.440657 (Hartree/Particle)

Thermal correction to Energy = 0.471907

Thermal correction to Enthalpy = 0.472852

Thermal correction to Gibbs Free Energy = 0.373132

Sum of electronic and zero-point Energies = -1564.187809

Sum of electronic and thermal Energies = -1564.156558

Sum of electronic and thermal Enthalpies = -1564.155614

Sum of electronic and thermal Free Energies = -1564.255333

$\mathbf{E_{sp} = -1564.25462}$ **(a.u.)**

atom	X/Å	Y/Å	Z/Å
C	5.48515	-5.20508	-0.2981
C	4.05475	-4.68755	-0.10343
C	3.90185	-3.16745	-0.09713
C	2.45382	-2.75886	0.10564
C	-4.75521	-1.39218	0.14338
C	-0.01498	-1.39667	0.47514
C	-3.60198	-0.69106	0.50803
C	-5.91354	-0.7041	-0.22471
C	-1.20664	-0.6931	0.6763
C	1.18517	-0.71078	0.27392
C	-5.90932	0.70445	-0.25379
C	-3.59861	0.7072	0.48123
C	-1.20382	0.7046	0.6501
C	1.18757	0.69895	0.24604
C	-4.74745	1.39989	0.08794
C	-0.01003	1.39581	0.42234
C	2.46395	2.73683	0.05958
C	3.91167	3.14986	-0.13644
C	4.05159	4.67119	-0.10307
C	5.47515	5.21438	-0.27052
O	5.62084	-6.46744	-0.41589

O	6.44645	-4.37668	-0.32418
N	-2.43063	-1.35445	0.95094
O	-7.09711	-1.31643	-0.58038
O	2.40532	-1.32042	0.0917
O	-7.08819	1.30905	-0.6373
N	-2.42463	1.38124	0.8995
O	2.40863	1.29896	0.03524
O	6.41744	4.41615	-0.56397
O	5.62317	6.47067	-0.10913
C	-7.13035	-2.7478	-0.56035
H	3.6882	-5.11087	0.84107
H	3.43495	-5.13211	-0.89161
H	4.26202	-2.75136	-1.04459
H	4.51075	-2.73189	0.70212
H	2.07142	-3.1262	1.06615
H	1.81554	-3.15099	-0.69612
H	-4.7353	-2.47533	0.16187
H	-0.03933	-2.47973	0.49023
H	-4.72103	2.48276	0.06334
H	-0.03183	2.4786	0.39494
H	1.82938	3.13877	-0.74032
H	2.07964	3.09657	1.02213
H	4.27322	2.7581	-1.09312
H	4.52317	2.69957	0.65339
H	3.65912	5.07	0.84022
H	3.44306	5.1252	-0.89641
H	-2.41617	-2.34358	0.72599
H	-2.40556	2.36133	0.63829
H	-8.13989	-3.02358	-0.86641
H	-6.40443	-3.16777	-1.26504
H	-6.93335	-3.13123	0.44676
C	-7.11755	2.74057	-0.65913
H	-8.12568	3.01004	-0.9753
H	-6.9214	3.15261	0.33678

H	-6.3892	3.13813	-1.37423

tr-dMeODBAP (1)

Zero-point correction = 0.440429 (Hartree/Particle)

Thermal correction to Energy = 0.471278

Thermal correction to Enthalpy = 0.472222

Thermal correction to Gibbs Free Energy = 0.373064

Sum of electronic and zero-point Energies = -1564.184082

Sum of electronic and thermal Energies = -1564.153233

Sum of electronic and thermal Enthalpies = -1564.152289

Sum of electronic and thermal Free Energies = -1564.251447

E_{sp} = -1564.245306 (a.u.)

atom	X/Å	Y/Å	Z/Å
C	6.67593	-3.67872	-0.23062
C	5.40789	-3.06779	-0.83782
C	4.39132	-2.52654	0.16668
C	3.18608	-1.94335	-0.54559
C	-4.8407	-1.29076	0.98975
C	-0.04665	-1.26047	0.71293
C	-3.63907	-0.75485	0.46008
C	-6.06138	-0.8079	0.56944
C	-1.34297	-0.71767	0.35119
C	1.08993	-0.90533	0.07601
C	-6.12609	0.24133	-0.40934
C	-3.70527	0.28952	-0.5046
C	-1.41253	0.35866	-0.59561
C	1.09698	0.08878	-1.07467
C	-4.96586	0.77305	-0.9314
C	-0.133	1.01053	-1.03851
C	2.7255	1.5074	0.07637
C	3.28205	2.88516	-0.24192
C	3.83943	3.55084	1.01534
C	4.43702	4.94962	0.82937
O	7.45685	-4.28292	-1.0369

O	6.89361	-3.54648	1.01308
N	-2.44014	-1.25285	0.87624
O	-7.27079	-1.25296	1.01545
O	2.26212	-1.47558	0.47793
O	-7.38733	0.63675	-0.74699
N	-2.56212	0.84305	-1.01476
O	2.28727	0.88	-1.14656
O	4.43508	5.48141	-0.32366
O	4.91003	5.50517	1.87508
C	-7.27254	-2.29622	2.00145
H	4.94693	-3.8304	-1.4767
H	5.73461	-2.26646	-1.514
H	4.85075	-1.74814	0.78497
H	4.06357	-3.32801	0.83811
H	2.66959	-2.70084	-1.14673
H	3.47253	-1.1082	-1.18796
H	-4.76914	-2.08404	1.72278
H	-0.01411	-2.03706	1.47047
H	-4.98958	1.56764	-1.66619
H	-0.26618	1.47447	-2.01779
H	1.89256	1.58627	0.78374
H	3.49466	0.87438	0.53496
H	2.48842	3.50148	-0.67869
H	4.07091	2.78778	-0.9966
H	4.62155	2.92332	1.46138
H	3.05522	3.63583	1.77872
H	-8.32243	-2.49087	2.21896
H	-6.79935	-3.20133	1.60772
H	-6.75735	-1.97003	2.91051
C	-7.51718	1.68636	-1.71768
H	-8.58898	1.84231	-1.8379
H	-7.04465	2.606	-1.35826
H	-7.07453	1.38704	-2.67307
H	1.09869	-0.48448	-2.00956

H	0.07408	1.81869	-0.32377

tr-dMeODBAP (2)

Zero-point correction = 0.440431 (Hartree/Particle)

Thermal correction to Energy = 0.471310

Thermal correction to Enthalpy = 0.472255

Thermal correction to Gibbs Free Energy = 0.372382

Sum of electronic and zero-point Energies = -1564.186852

Sum of electronic and thermal Energies = -1564.155972

Sum of electronic and thermal Enthalpies = -1564.155028

Sum of electronic and thermal Free Energies = -1564.254901

E_{sp} = -1564.249018 (a.u.)

atom	X/Å	Y/Å	Z/Å
C	6.39332	3.51294	0.5432
C	5.02743	2.94253	0.94163
C	4.20948	2.32306	-0.1903
C	2.87221	1.82344	0.32057
C	-5.05488	1.26156	-0.52436
C	-0.25038	1.29956	-0.65319
C	-3.77011	0.63663	-0.26502
C	-6.20544	0.5496	-0.45818
C	-1.47258	0.64954	-0.35087
C	0.94813	0.65658	-0.45754
C	-6.20865	-0.93803	-0.13708
C	-3.72679	-0.68677	0.30111
C	-1.43416	-0.67452	0.1727
C	0.98642	-0.68177	0.05113
C	-5.01813	-1.30586	0.75417
C	-0.19535	-1.32954	0.36424
C	2.34802	-2.567	0.67544
C	3.82315	-2.91948	0.7181
C	4.4765	-2.92893	-0.66315
C	5.95013	-3.34543	-0.70326
O	7.0718	4.05459	1.4773

O	6.77956	3.42488	-0.66272
N	-2.65612	1.29207	-0.56843
O	-7.43431	1.01587	-0.76765
O	2.13127	1.27624	-0.80598
O	-7.45182	-1.3849	0.40031
N	-2.59584	-1.3235	0.50569
O	2.22759	-1.21632	0.17786
O	6.54964	-3.62893	0.38
O	6.49664	-3.37908	-1.85494
C	-7.55632	2.3956	-1.15376
H	4.46492	3.75646	1.41619
H	5.2056	2.20198	1.73174
H	4.75701	1.48656	-0.63809
H	4.03903	3.06346	-0.97971
H	2.27886	2.6376	0.7546
H	3.00428	1.04235	1.07629
H	-5.0411	2.29158	-0.8587
H	-0.26073	2.31026	-1.0474
H	-5.20882	-0.93007	1.76827
H	1.90263	-2.61649	1.67519
H	1.79639	-3.23994	0.00784
H	3.90374	-3.91073	1.17776
H	4.34083	-2.21813	1.38188
H	4.41204	-1.93612	-1.12301
H	3.92901	-3.6051	-1.3325
H	-8.61895	2.55751	-1.33099
H	-7.20474	3.0485	-0.34921
H	-6.98825	2.58804	-2.06895
H	-0.20368	-2.33943	0.75395
H	-4.91863	-2.39139	0.81521
H	-6.12728	-1.47156	-1.09227
C	-7.79766	-0.86906	1.69377
H	-8.86532	-1.05881	1.82025
H	-7.24744	-1.38176	2.48985

H	-7.61456	0.20866	1.76136

Supplementary Table 3. Comparsion of OCV at 100 % SOC of this work and previous reported alkaline AOFBs.

Redox-active molecules	OCV at 100 % SOC (V)
**dMeODBAP (this work)**	**1.39**
2,6-DHAQ[5]	1.34
2,6-DBEAQ[6]	1.12
2,6-DPPEAQ[7]	1.05 (at 90 % SOC)
DAEAQ[8]	1.21
AQ-1,8-3E-OH[9]	1.1
PEG12-AQ[10]	1.01
DCDHAQ[11]	1.23 (at 90 % SOC)
DPivOHAQ[12]	1.08
2,6-D2PEAQ[13]	1.34
Cys-AE[15]	1.12
DHPS[17]	1.48
BHPC[18]	1.36
1,6-DPAP[19]	1.19
1,8-PFP[20]	1.24
2,3-O-DBAP[21] (DBEP[22])	1.28

Supplementary Table 4. Cost calculation of dMeODBAP.

Species	Cost ($ g^{-1})	Molar weight (g mol^{-1})	Reaction equivalent
1,2-Dimethoxybenzene	0.03	138.16	1
Nitric acid	0.0047	63.01	2
Hydrazine hydrate (85 %)	0.05	32.05	15
2,5-Dihydroxy-1,4-benzoquinone	1.4	140.09	1
Methyl 4-bromobutanoate	0.171	181.03	4.5
dMeODBAP*	1.64	444	/

*The overall yield of dMeODBAP is 55 %.

REFERENCES

1. K. Mizrahi Rodriguez, A. X. Wu, Q. Qian, G. Han, S. Lin, F. M. Benedetti, H. Lee, W. S. Chi, C. M. Doherty and Z. P. Smith, Facile and Time-Efficient Carboxylic Acid Functionalization of PIM-1: Effect on Molecular Packing and Gas Separation Performance, *Macromolecules*, 2020, **53**, 6220-6234.
2. X. Weng, J. E. Baez, M. Khiterer, M. Y. Hoe, Z. Bao and K. J. Shea, Chiral Polymers of Intrinsic Microporosity: Selective Membrane Permeation of Enantiomers, *Angewandte Chemie International Edition*, 2015, **54**, 11214-11218.
3. A. Wang, R. Tan, D. Liu, J. Lu, X. Wei, A. Alvarez-Fernandez, C. Ye, C. Breakwell, S. Guldin, A. R. Kucernak, K. E. Jelfs, N. P. Brandon, N. B. McKeown and Q. Song, Ion-Selective Microporous Polymer Membranes with Hydrogen-Bond and Salt-Bridge Networks for Aqueous Organic Redox Flow Batteries, *Advanced Materials*, 2023, **35**, 2210098.
4. C. Ye, R. Tan, A. Wang, J. Chen, B. Comesaña Gándara, C. Breakwell, A. Alvarez-Fernandez, Z. Fan, J. Weng, C. G. Bezzu, S. Guldin, N. P. Brandon, A. R. Kucernak, K. E. Jelfs, N. B. McKeown and Q. Song, Long-Life Aqueous Organic Redox Flow Batteries Enabled by Amidoxime-Functionalized Ion-Selective Polymer Membranes, *Angewandte Chemie International Edition*, 2022, **61**, e202207580.
5. K. Lin, Q. Chen, M. R. Gerhardt, L. Tong, S. B. Kim, L. Eisenach, A. W. Valle, D. Hardee, R. G. Gordon, M. J. Aziz and M. P. Marshak, Alkaline quinone flow battery, *Science*, 2015, **349**, 1529-1532.
6. D. G. Kwabi, K. Lin, Y. Ji, E. F. Kerr, M.-A. Goulet, D. De Porcellinis, D. P. Tabor, D. A. Pollack, A. Aspuru-Guzik, R. G. Gordon and M. J. Aziz, Alkaline Quinone Flow Battery with Long Lifetime at pH 12, *Joule*, 2018, **2**, 1894-1906.
7. Y. Ji, M.-A. Goulet, D. A. Pollack, D. G. Kwabi, S. Jin, D. De Porcellinis, E. F. Kerr, R. G. Gordon and M. J. Aziz, A Phosphonate-Functionalized Quinone Redox Flow Battery at Near-Neutral pH with Record Capacity Retention Rate, *Advanced Energy Materials*, 2019, **9**, 1900039.
8. C. Wang, B. Yu, Y. Liu, H. Wang, Z. Zhang, C. Xie, X. Li, H. Zhang and Z. Jin, N-alkyl-carboxylate-functionalized anthraquinone for long-cycling aqueous redox flow batteries, *Energy Storage Materials*, 2021, **36**, 417-426.
9. S. Jin, Y. Jing, D. G. Kwabi, Y. Ji, L. Tong, D. De Porcellinis, M.-A. Goulet, D. A. Pollack, R. G. Gordon and M. J. Aziz, A Water-Miscible Quinone Flow Battery with

High Volumetric Capacity and Energy Density, *ACS Energy Letters*, 2019, **4**, 1342-1348.

10. J. Chai, X. Wang, A. Lashgari, C. K. Williams and J. Jiang, A pH-Neutral, Aqueous Redox Flow Battery with a 3600-Cycle Lifetime: Micellization-Enabled High Stability and Crossover Suppression, *ChemSusChem*, 2020, **13**, 4069-4077.
11. M. Wu, M. Bahari, E. M. Fell, R. G. Gordon and M. J. Aziz, High-performance anthraquinone with potentially low cost for aqueous redox flow batteries, *Journal of Materials Chemistry A*, 2021, **9**, 26709-26716.
12. M. Wu, Y. Jing, A. A. Wong, E. M. Fell, S. Jin, Z. Tang, R. G. Gordon and M. J. Aziz, Extremely Stable Anthraquinone Negolytes Synthesized from Common Precursors, *Chem*, 2020, **6**, 1432-1442.
13. E. F. Kerr, Z. Tang, T. Y. George, S. Jin, E. M. Fell, K. Amini, Y. Jing, M. Wu, R. G. Gordon and M. J. Aziz, High Energy Density Aqueous Flow Battery Utilizing Extremely Stable, Branching-Induced High-Solubility Anthraquinone near Neutral pH, *ACS Energy Letters*, 2023, **8**, 600-607.
14. Y. Jing, E. M. Fell, M. Wu, S. Jin, Y. Ji, D. A. Pollack, Z. Tang, D. Ding, M. Bahari, M.-A. Goulet, T. Tsukamoto, R. G. Gordon and M. J. Aziz, Anthraquinone Flow Battery Reactants with Nonhydrolyzable Water-Solubilizing Chains Introduced via a Generic Cross-Coupling Method, *ACS Energy Letters*, 2022, **7**, 226-235.
15. Y. Liu, Z. Wu, P. Zhang, J. Wei, J. Li, H. Wang, S. Wen, J. Liang, Y. Chen, T. Dai, Z. Tie, J. Ma, X. Wang and Z. Jin, Artificial α-amino acid based on cysteine grafted natural aloe-emodin for aqueous organic redox flow batteries, *Nature Communications*, 2025, **16**, 2965.
16. P. Zhang, Y. Liu, J. Wei, Z. Wu, X. Song, G. Ding, H. Wang, J. Liang, Z. Tie and Z. Jin, An amphoteric and hydrogen-bond-rich artificial α-amino acid for highly durable aqueous redox flow batteries, *Nature Communications*, 2025, **16**, 4727.
17. A. Hollas, X. Wei, V. Murugesan, Z. Nie, B. Li, D. Reed, J. Liu, V. Sprenkle and W. Wang, A biomimetic high-capacity phenazine-based anolyte for aqueous organic redox flow batteries, *Nature Energy*, 2018, **3**, 508-514.
18. C. Wang, X. Li, B. Yu, Y. Wang, Z. Yang, H. Wang, H. Lin, J. Ma, G. Li and Z. Jin, Molecular Design of Fused-Ring Phenazine Derivatives for Long-Cycling Alkaline Redox Flow Batteries, *ACS Energy Letters*, 2020, **5**, 411-417.

19. S. Pang, X. Wang, P. Wang and Y. Ji, Biomimetic Amino Acid Functionalized Phenazine Flow Batteries with Long Lifetime at Near-Neutral pH, *Angewandte Chemie International Edition*, 2021, **60**, 5289-5298.
20. J. Xu, S. Pang, X. Wang, P. Wang and Y. Ji, Ultrastable aqueous phenazine flow batteries with high capacity operated at elevated temperatures, *Joule*, 2021, **5**, 2437-2449.
21. T. Kong, J. Li, W. Wang, X. Zhou, Y. Xie, J. Ma, X. Li and Y. Wang, Enabling Long-Life Aqueous Organic Redox Flow Batteries with a Highly Stable, Low Redox Potential Phenazine Anolyte, *ACS Applied Materials & Interfaces*, 2024, **16**, 752-760.
22. Y. Liu, P. Zhang, Z. Wu, J. Wei, G. Ding, X. Song, J. Ma, W. Wang and Z. Jin, Screening Ultra-Stable (Phenazine)dioxyalkanocic Acids with Varied Water-Solubilizing Chain Lengths for High-Capacity Aqueous Redox Flow Batteries, *Journal of the American Chemical Society*, 2024, **146**, 3293-3302.
23. Y. Cui, K. Zheng, R. Sun, Z. Yuan, D. Guo, J. Xu, X. Yu, J. Zang and J. Cao, A multi-substituted phenazine derivative aqueous redox flow battery with high energy efficiency and long lifetime, *Journal of Power Sources*, 2025, **633**, 236461.
24. L. Li, E. Yao, Y. Ji and P. Wang, A Resonance Hybrid Design for Stable Aqueous Organic Redox Flow Batteries, *Angewandte Chemie International Edition*, 2025, **n/a**, e202423219.
25. R. Feng, X. Zhang, V. Murugesan, A. Hollas, Y. Chen, Y. Shao, E. Walter, N. P. N. Wellala, L. Yan, K. M. Rosso and W. Wang, Reversible ketone hydrogenation and dehydrogenation for aqueous organic redox flow batteries, *Science*, 2021, **372**, 836-840.
26. S. Singh, J. L. Tami, C. Gruich, A. J. Gatz, J. Dong, B. H. Nguyen, J. A. Smith, B. R. Goldsmith, A. J. McNeil and D. G. Kwabi, Sulfonated Benzo[c]cinnolines for Alkaline Redox-Flow Batteries, *ACS Applied Energy Materials*, 2025, **8**, 7904-7911.
